# Distribution of new satellites and simple sequence repeats in annual and perennial *Glycine* species

**DOI:** 10.1186/s40529-015-0103-9

**Published:** 2015-09-16

**Authors:** Hsuan Chen, Mei-Chu Chung, Yuan-Ching Tsai, Fu-Jin Wei, Jaw-Shu Hsieh, Yue-Ie C. Hsing

**Affiliations:** 1 Institute of Plant and Microbial Biology, Academia Sinica, Taipei, 115 Taiwan; 2 Department of Agronomy, National Taiwan University, Taipei, 106 Taiwan

**Keywords:** *Glycine*, FISH, SSRs, 45S rDNA, Repeat sequence, Chromosome markers

## Abstract

The repeat sequences occupied more than 50 % of soybean genome. In order to understand where these repeat sequences distributed in soybean genome and its related *Glycine* species, we examined three new repeat sequences—soybean repeat sequence (SBRS1, SBRS2 and SBRS3), some nonspecific repeat sequences and 45S rDNA on several *Glycine* species, including annual and perennial accessions in this study. In the annual species, *G. soja*, signals for SBRS1 and ATT repeat can be found on each chromosome in GG genome, but those for SBRS2 and SBRS3 were located at three specific loci. In perennial *Glycine* species, these three SBR repeat frequently co-localized with 45S rDNA, two major 45S rDNA loci were found in all tetraploid species. However, an extra minor locus was found in one accession of the *G. pescadrensis* (Tab074), but not in another accession (Tab004). We demonstrate that some repetitive sequences are present in all *Glycine* species used in the study, but the abundancy is different in annual or perennial species. We suggest this study may provide additional information in investigations of the phylogeny in the *Glycine* species.

**Electronic supplementary material:**

The online version of this article (doi:10.1186/s40529-015-0103-9) contains supplementary material, which is available to authorized users.

## Background

Soybean (*Glycine max* L. Merrill) is the most important legume crop because of its economic value and biological features for research. Since soybean seed consists of high protein and oil contents, it has become one of the most important food crops for human and livestock, as well as a valuable resource for biofuel energy. Improving soybean varieties has been slow and limited because of the lack of genetic diversity (Keim et al. 1990; Krishnan et al. 2001). Wild germplasm may be a significant breeding resource for current varieties that have undergone several genetic bottlenecks and have shown limited genetic variability (Hyten et al. 2006; Keim et al. 1990). These wild germplasm harbor many traits of interest to soybean breeders, such as biotic and abiotic disease resistance (Newell and Hymowitz 1975), high seed protein content (Sebolt et al. 2000) and increased yield (Concibido et al. 2003). Thus, investigating *Glycine* species is important for soybean genetics and breeding.

There are around 30 species in Genus *Glycine* which had been classified into two subgenera, *Soja* and *Glycine*. The subgenus *Soja* (2n = 40) includes only *G. max* and its annual close related *G. soja* Sieb. and Zucc., which may intercross freely with soybean (Palmer et al. 1987; Singh and Hymowitz 1988). The subgenus *Glycine* contains all perennial species with various genome types and ploidies (2n = 2x = 40, 2n = 4x = 80 and some aneuploids) (Doyle et al. 2004; Grant et al. 1984; Newell and Hymowitz 1983; Singh and Hymowitz 1985). Early investigations of phylogenetic relationships in *Glycine* species based on their ability to produce fertile hybrids and the degree of meiotic chromosomes pairing (reviewed by Ratnaparkhe et al. 2011). This led to the designation of seven genome groups, i.e. reproductively compatible within species, while reproductively isolated from other genome groups (groups A to G, Singh and Hymowitz 1985; Hymowitz et al. 1998). Other data, including isozymes (Doyle et al. 1986) and DNA phylogenetic analyses (Doyle et al. 1990a, b, c; Kollipara et al. 1997), suggested hybrid fertility as the primary criterion to identify species to genome groups and resulted in the designation of two additional genome groups, H and I. Besides, the situation in some species even more complicated, such as the *G. tabacina* and *G. tomentella* species complex, multiple separate ‘races’ are recognized that have not yet taxonomically arisen to species status, but that display enough molecular phylogenetic divergence from one another to be grouped into separate taxa (Doyle et al. 2004; Sherman-Broyles et al. 2014).

The *Glycine* species germplasm in Taiwan is abundant and important. Four species have been collected, including *G. soja* Siebold and Zucc. (GG genome, 2n = 40), *G. tomentella* Hayata (DDD_1_D_1_ genome, 2n = 80), *G. dolichocarpa* Tateishi and Ohashi (A_6_A_6_DD genome, 2n = 80) and *G. pescadrensis* Hayata (A_6_A_6_B_3_B_3_ genome, 2n = 80) (Hsing et al. 1995, 2001; Tateishi and Ohashi 1992; Thseng et al. 1999; Tsai et al. 2001; Tsai 2006). *G. soja* is widely distributed in Russia, Korea, Japan, China and Taiwan (Singh and Hymowitz 1987), with Taiwan being the southernmost among these areas. The three wild perennial *Glycine* species, *G. tomentella, G. dolichocarpa* and *G. pescadrensis*, are found in Taiwan, the Ryukyu Islands, the Philippines, the South Pacific islands and Australia, with Taiwan being the northernmost of these areas (Hymowitz et al. 1998). All the remaining perennial *Glycine* species are found only in Australia (Tindale and Craven 1988). Therefore, the Ryukyu Islands, Taiwan, and its adjacent islands are unusual because they have representatives of both subgenera of *Glycine*. The collection and study of *Glycine* species in Taiwan and its adjacent islands thus provide information for better understanding the evolution and relationships between these two subgenera (Hsieh et al. 2001).

The soybean genome has been well documented as paleopolyploidy and manifested as allopolyploid diploidy (Gill et al. 2009; Mudge et al. 2005; Schlueter et al. 2007; Shoemaker et al. 1996; Tek et al. 2010). In addition, whole-genome sequencing (Williams 82) and resequencing projects on *Glycine* species were available recently (Jackson et al. 2006; Katayose et al. 2012; Kim et al. 2010; Lam et al. 2010; Schmutz et al. 2010; Weidner et al. 2012). However, little information is available on the genome composition of wild relatives; that is, cytological research of wild soybean is still limited.

Fluorescent in situ hybridization (FISH) is a powerful tool for cytological studies. FISH used with specific markers can be used to label specific chromosomes and target given genomes or particular regions on chromosomes (Cheng et al. 2007; Jiang and Gill 2006; Kopecky et al. 2008; Pinkel et al. 1986). Furthermore, nonspecific repeats such as simple repeat sequences (SSRs) may also be used as genomic and chromosomal markers among many species (Cuadrado et al. 2008; Cuadrado and Jouve 2007).

FISH has been used in cytological research on soybean. Several repeat sequences such as ribosomal RNA genes (rDNAs) (Shi et al. 1996), SB92 (Kolchinsky and Gresshoff 1995; Vahedian et al. 1995) and STR120 (Morgante et al. 1997) were labeled on soybean chromosomes and used in chromosome “painting” (Shi et al. 1996). FISH with bacterial artificial chromosome (BAC) clones used as probes suggested heterochromatic blocks on the chromosome pericentromeric region (Lin et al. 2005), and some research has provided evidence of chromosome-level homeology in the paleopolyploid soybean genome (Pagel et al. 2004; Walling et al. 2006). In the study of soybean chromosome structure and genomic evolution, SB92 was previously suggested as a major repeat sequence in the *G. max* genome (Kolchinsky and Gresshoff 1995; Vahedian et al. 1995). Later, SB92 and its similar sequence SB91 were both defined as the centromere sequences of the GG genome by cytological features (Gill et al. 2009) and thus designated CentGm-1 (SB92) and CentGm-2 (SB91) (Tek et al. 2010). In addition, FISH revealed CentGm-1 or CentGm-2 present in about a half of all *G. max* chromosomes, which suggests allopolyploidy in the paleopolyploid genome (Gill et al. 2009). The *G. max* karyotyping system in mitotic metaphase was recently studied with a probe cocktail of BAC clones and partial centromere oligonucleotides (Findley et al. 2010), and this system has enabled the characterization of most of the chromosomal translocation lines (Findley et al. 2011).

The rDNAs have been widely used as reliable landmarks in chromosome karyotyping of several higher plants. In higher eukaryotes, the 45S and 5S rDNA loci are transcribed by different RNA polymerases and usually located in different positions on chromosomes (Srivastava and Schlessinger 1991). The number and location on chromosomes for both 45S and 5S may be diverse within related species (Chang et al. 2009; Chung et al. 2008; Pedrosa-Harand et al. 2006). In other words, karyotyping with rDNA loci may reflect the relationships of related species. Most of the recent cytological research on *Glycine* species has focused on the number of rDNA loci. The genome from diploid *Glycine* species, known as the paleopolyploidy, was predicted to consist of two 45S rDNA loci because it was ancient allopolyploidy; however, only one locus was observed on diploid *Glycine* species (Krishnan et al. 2001; Singh et al. 2001). Therefore, one 45S rDNA locus disappeared during soybean genome diploidization, whereby the ancient tetraploid became a new diploid paleopolyploidy.

Some studies revealed two 5S rDNA and two 45S rDNA loci in most allotetraploid genomes (Krishnan et al. 2001; Singh et al. 2001); however, an extra minor 45S rDNA locus was found in only two allotetraploid species (Krishnan et al. 2001). Therefore, the number of 45S loci among *Glycine* species or accessions was not as simple as the model of “diploid-doubled to tetraploid” of one locus for diploid and two loci for tetraploid species (Krishnan et al. 2001; Singh et al. 2001).

In the current study, we investigated the difference in repeat sequences between soybean and its wild related species. Here, we report three new soybean repeat sequences—SBRS1, SBRS2 and SBRS3—isolated from the soybean WSG data. In light of their distribution on chromosomes of GG genome, the three repeat sequences may be considered as FISH karyotyping markers for GG genome. We also labeled the three sequences in some wild *Glycine* species and found that they widely co-localized with 45S rDNA.

## Methods

### Plant materials

*Glycine* species used and locations where they were collected are in Table 1 along with their genome types and known USDA permanent plant introduction (PI) numbers or IL number designated by Dr. T. Hymowitz. All plant materials were previously described in detail (Hsieh et al. 2001). Cultivated soybean Shi-shi was kindly provided by Kaohsiung District Agricultural Research and Extension Station. *Glycine* species distributed in Taiwan and nearby islands were collected by Drs. J. S. Hsieh and Y. C. Huang, Department of Agronomy, National Taiwan University. Three diploid *Glycine tomentella* accessions originally from Papua New Guinea and Australia were kindly provided by Dr. T. Hymowitz, Department of Crop Science, University of Illinois, USA. These plants were grown in the greenhouse at Academia Sinica, Taiwan. Root tips of seedling were used for metaphase chromosome preparation.

**Table 1:** The accessions of the *Glycine species* used in this study, with their nuclear DNA content and collection locations

Species	Genome types	Accession numbers		2C DNA content		Collection location
		TW^a^	IL or PI^c^	PG^b^	Mbp^b^	
*G. max*	GG	Shi-shi	PI416967	2.44 ± 0.01	2360	Cultivar
*G. soja*	GG	Soja001	PI393551	2.00 ± 0.02	1930	Shimen, Taoyuan, Taiwan
*G. syndetika*	A_6_A_6_	Tom052	PI441000	2.58 ± 0.00	2490	North Queensland, Australia
*G. tomentella*	DD	Tom062	PI446993	2.11 ± 0.01	2040	Papua New Guinea
*G. tomentella*	D_1_D_1_	Tom051	PI505301	2.24 ± 0.10	2170	West Australia
*G. pescadrensis*	A_6_A_6_B_3_B_3_	Tab004	IL875	4.52 ± 0.02	4370	Houliao, Penghu, Taiwan
*G. pescadrensis*	A_6_A_6_B_3_B_3_	Tab074	–	4.90 ± 0.03	4730	Kingmen, Taiwan
*G. dolichocarpa*	A_6_A_6_DD	Tom039	PI320548	5.17 ± 0.05	4990	Tungho, Taitung, Taiwan
*G. tomentella*	DDD_1_D_1_	Tom034	IL872	5.18 ± 0.21	5000	Daguang, Pintung, Taiwan

^a^Accession number in our laboratory

^b^1 pg DNA = 965 Mbp (Bennett et al. 2000), N = 3

^c^Illinois (IL) numbers or USDA Plant introduction (PI) numbers

### Flow cytometry for estimating ploidy

Flow cytometric estimation of nuclear DNA content involved the MoFlo XDP Cell Sorter (Beckman Coulter, Fullerton, CA, USA), with propidium iodide (PI) as the fluorescent stain. A total of 20 mg of young leaves of each plant and standard nuclear fractions were prepared and mixed. Chicken erythrocyte nuclei (CEN; BioSure Inc., Grass Valley, CA, USA) and rainbow trout erythrocyte nuclei (TEN; NPE Systems Inc, Pembroke Pines, FL, USA) were used as internal standards and the DNA index (Vindelov et al. 1983). Leaf samples were chopped by using of a razor in a Petri dish containing 0.5 ml extraction buffer (Partec CyStain PI Absolute P Nuclei Extraction Buffer; Partec GMBH, Munster, Germany). The resulting extract was passed through two 30-μm filters before the addition of 0.5 ml Partec CyStain PI Absolute P Staining Buffer. Samples were kept in the dark for up to 30 min before flow cytometry. Six thousand cells were counted in each sample, with three replicates. The predicted genome size was calculated by assuming that 1 pg DNA = 965 Mbp (Bennett et al. 2000).

### Predictions of repeat sequences in the soybean genome

Candidate repeat sequences were identified on the basis of the soybean WGS data (Schmutz et al. 2010). “eTandem” in EMBOSS 6.0 was used to scan all scaffold sequences, and the outputs were filtered by shell scripts and AWK script in Linux with repeat unit length >70 bp, homologous identity >70 % and copies >30. The longest repeats were used for primer design (Additional file 1: Table S1).

### Syntheses of probes for FISH analysis

To prepare probes for FISH analysis, the pTA71 plasmid that contains a 9.1-kb fragment of 18S-5.8S-26S rDNA from common wheat (Gerlach and Bedbrook 1979) was labeled with biotin-16-dUTP by nick translation (Roche Diagnostics, Penzberg, Germany). Other repeat sequences were subcloned into pGEM-T Easy vector (Promega) by using Shi-shi genomic DNA and were labeled with digoxigenin-11-dUTP by using the Roche PCR DIG Probe synthesis kit (Roche).

### Southern blot analysis

Genomic DNA (30 μg) of each *Glycine* accession was completely digested with restriction enzymes (*Eco*RI or *Nde*I, New England Biolabs) before separation by electrophoresis overnight on a 0.8 % agarose gel in 0.5X TBE buffer, then blotting onto Hybond-N+ nylon membranes (Amersham Pharmacia Biotech). Probe preparation, membrane hybridization, and signal detection followed the instructions of the ECL direct nucleic acid labeling and detection system (Amersham Pharmacia Biotech).

### Chromosome preparation and FISH analysis

The terminal 0.5 cm of young roots was collected from individual seedlings, pretreated with 2 mM 8-hydroxyquinoline for 2 h at room temperature and fixed overnight in Farmer’s fixative [95 % ethanol + glacial acetic acid (3:1 v/v)]. The fixed root tips were washed with distilled water before incubation in a cell wall digestive enzyme mixture of 6 % pectinase (Sigma Chemical, St. Louis, MO, USA) and 6 % cellulase (Onozuka R-10; Yakult Honsha, Tokyo, Japan) in 75 mM KCl (pH = 4.0) buffer at 37 °C for 1.5–3 h depending on the characteristics of each accession.

After a wash with distilled water, the terminal 1 mm of softened tissues was squashed on a slide (Chang et al. 2009). Samples were pretreated with pepsin (1.25 mg/ml for *G. soja*, 5 mg/ml for other accessions, in 10 mM HCl) at 37 °C for 3 to 8 h depending on the characteristics of each accession. FISH analysis was performed as described previously (Chung et al. 2008).

## Results

### Estimation of genome size

The DNA contents of the *Glycine* species were analyzed by flow cytometry (Table 1). The estimated 2C DNA contents of the diploid species, cultivated soybean Shi-shi (*Glycine max*, GG genome), Soja001 (*Glycine soja*, GG genome), Tom052 (*Glycine syndetike*, A_6_A_6_ genome), Tom062 (*Glycine tomentella*, DD genome), and Tom051 (*Glycine tomentella*, D_1_D_1_ genome), were 2360, 1930, 2500, 2040 and 2170 Mb, respectively, and contents varied between different species by 29 %. The estimated 2C DNA contents of the tetraploid species, Tab 004 (*G. pescadrensis*, A_6_A_6_B_3_B_3_ genome), Tab 074 (*G. pescadrensis*, A_6_A_6_B_3_B_3_ genome), Tom039 (*Glycine dolichocarpa*, A_6_A_6_DD genome) and Tom034 (*Glycine tomentella*, DDD_1_D_1_ genome), were 4370, 4730, 4990 and 5000 Mb, respectively, and differed between different species or accessions by 15 %. The DNA for two *G. pescadrensis* accessions, Tab 004 and Tab 074, was collected from Penghu and Kingmen islands, respectively and the content differed by 9 %. When compare the tetraploid with its two diploid parental species, the DNA content of *G. dolichocarpa* (Tom039) is higher than the total DNA content from *G. syndetike* (Tom052) and *G. tomentella* (Tom062), the DNA content of tetraploid *G. tomentella* (Tom034) is higher than the total DNA content from two diploid *G. tomentella* (Tom051 and Tom062).

### Repeat sequences from WGS data and the analysis

To investigate the repeat sequence component in the *Glycine* genome, we used the WGS data to find new repeat sequences. We identified 17 candidate repeat sequences by bioinformatics analysis. Several of these candidates belong to the LTR120 family (Morgante et al. 1997) and SB92 family (Kolchinsky and Gresshoff 1995). Finally, we chose 3 new repeat sequences, designated soybean repeat sequence1 (SBRS1), SBRS2 and SBRS3; their sequences and designed primers are in Additional files 1 and 2.

#### SBRS1

A 48 bp minisatellite sequence, represents approximately 131,000 copies in the WGS data; 123,000 copies were aligned into 13 pseudomolecules, including Gm1, 2, 3, 5, 11, 12, 14, 15, 16, 17, 18, 19 and 20. The remaining copies were aligned into contigs. SBRS1 is a head-to-tail tandem repeat with tens to hundreds of copies in each region.

#### SBRS2

A 124 bp minisatellite sequence, represents approximately 6000 copies in the WGS data. About 95 % of the SBRS2 repeats were aligned into pseudomolecules. The SBRS2 is a head-to-tail tandem repeat with usually less than 10 copies in each continguous region and is dispersed on all of the 20 pseudomolecules in the WSG data. The most significant region rich in SBRS2 was located at Gm9, where 1095 copies of the SBRS2 clustered in a 270-kb region.

#### SBRS3

A 201 bp minisatellite sequence, represents 8206 copies in the WGS data; 8016 copies were aligned into pseudomolecules. It is also a tandem repeat, with usually less than 10 copies in each region, dispersed on all 20 pseudomolecules. Three regions especially contain a high density of SBRS3 repeats, including 2 on Gm14 (1512 copies were separated into 2 groups 26,000 kb apart) and the other one on Gm20 (400 copies clustered together).

**Fig. 1:**
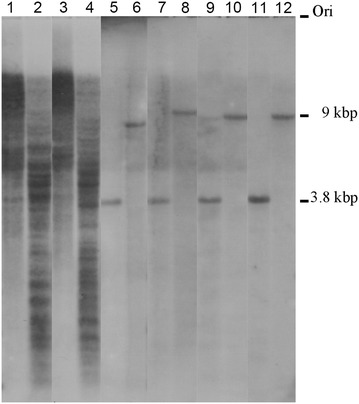
Southern blot analysis of the SBRS1 on *Glycine* species

To understand the existence and the distribution of these three repeat sequences in the *Glycine* genome, we used them as probes in Southern blot analyses with genomic DNA of the cultivated soybean and the collected *Glycine* species. All three repeats, SBRS1, SBRS2 and SBRS3, showed heavy ladder signals in the GG genome species, *G. max* and *G. soja* (Lanes 1–4 of Fig. 1; Additional file 3: Figure S1, Additional file 4: Figure S2). These heavy ladder signals are similar between *G. max* and *G. soja*, but the patterns among the three repeat sequences are different. However, the 3 repeat sequences showed single band and the similar size in other genome species, including *G. tomentella*, *G. dolichocarpa* and *G. pescadrensis*, a 3.8- and a 9-kbp band were found in *Eco*RI- and *Nde*I-digested DNA samples, respectively, of all perennial *Glycine* species tested (Lanes 5–12 of Fig. 1. Additional file 3: Figure S1, Additional file 4: Figure S2). Therefore, the three repeat sequences exist in each *Glycine* species tested, with high copies in GG genome, but with very low copies in other species.

### FISH analysis with SBRS1, SBRS2, SBRS3 and 45S rDNA

**Fig. 2:**
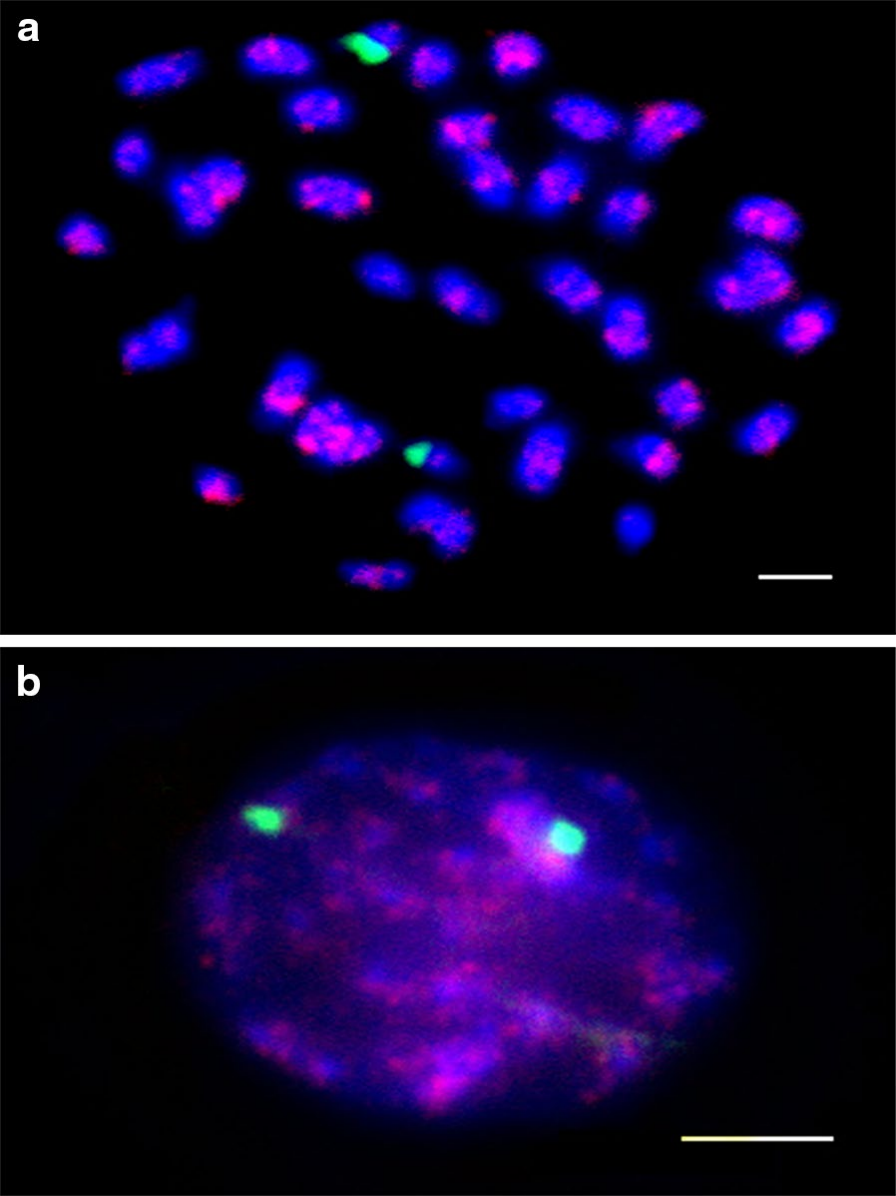
FISH analysis of SBRS1 and 45S rDNA on *G. soja* chromosomes

FISH analysis was used in further evaluated the distribution of the three repeat sequences in the genome of *Glycine* species. We used the root tips of *G. soja* to represent the GG genome on FISH analysis because the cultivated soybean and *G. soja* both represent the GG genome, and band patterns on Southern blot analysis were similar (Fig. 1, Additional file 3: Figure S1, Additional file 4: Figure S2). The 45S rDNA probe was used as a control, and it was located at the distal ends of one pair of chromosomes of *G. soja* (Fig. 2a, green).

**Fig. 3:**
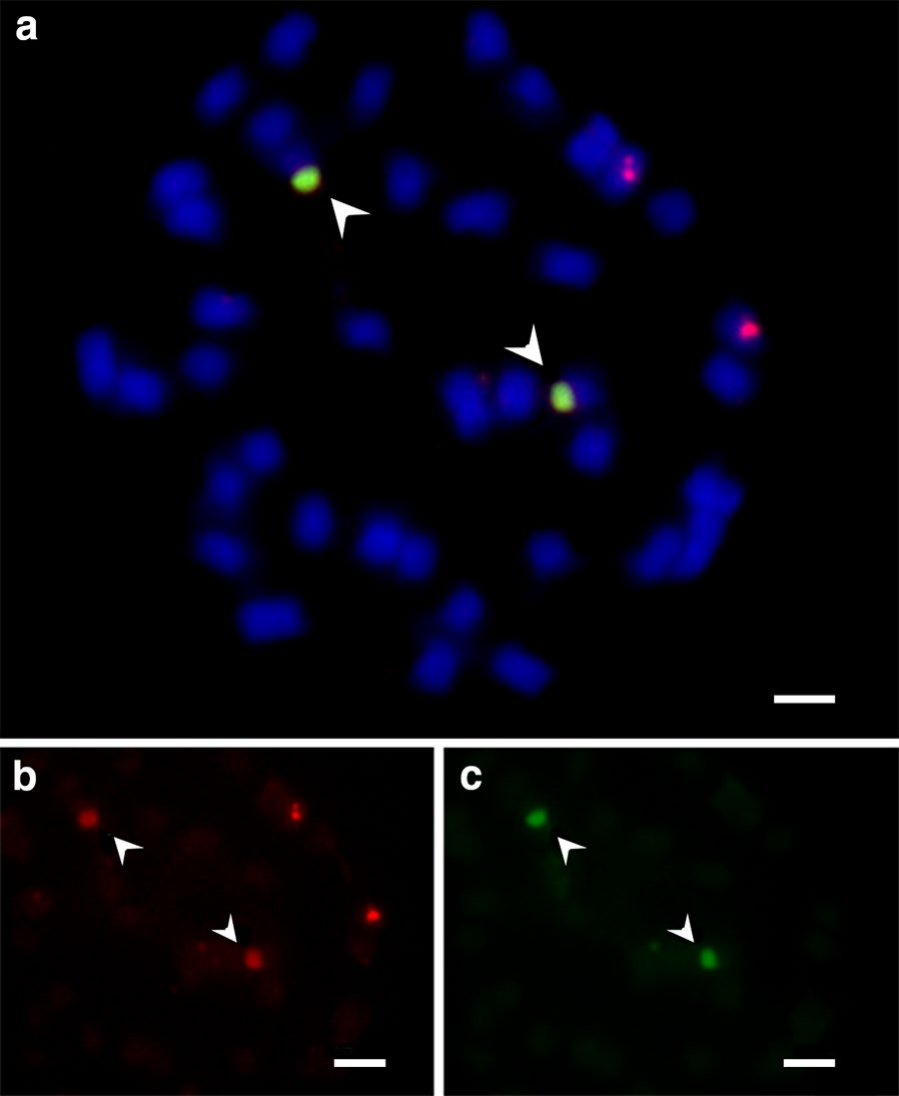
FISH analysis of SBRS2 and 45S rDNA on *G. soja* chromosomes

**Fig. 4:**
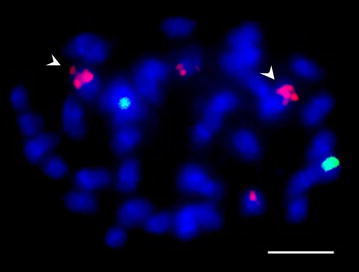
Localization of SBRS3 and 45S rDNA on *G. soja* mitotic chromosomes

SBRS1 signals were dispersed on every chromosome of *G. soja* as shown by metaphase (Panel A) and interphase (Panel B) chromosomes (Fig. 2a, red). Throughout the genome, the distribution of SBRS1 signals varied among chromosomes. SBRS2 signals were detected as clusters on two pairs of chromosomes of *G. soja* (Fig. 3). FISH signals for one of the SBRS2 clusters overlapped with 45S rDNA at the distal ends of one pair of chromosomes (Fig. 3a, yellowish green), and the second SBRS2 cluster was detected in the proximal regions of another pair of chromosomes (Fig. 3a, b, red). SBRS3 signals were detected on three pairs of chromosomes of *G. soja* (Fig. 4, red), and none overlappped with 45S rDNA (Fig. 4, green). One of the SBRS3 signals was detected at the sub-distal ends of one pair of chromosomes, and the other 2 were detected in the proximal regions and the distal ends of another pair of chromosomes.

**Fig. 5:**
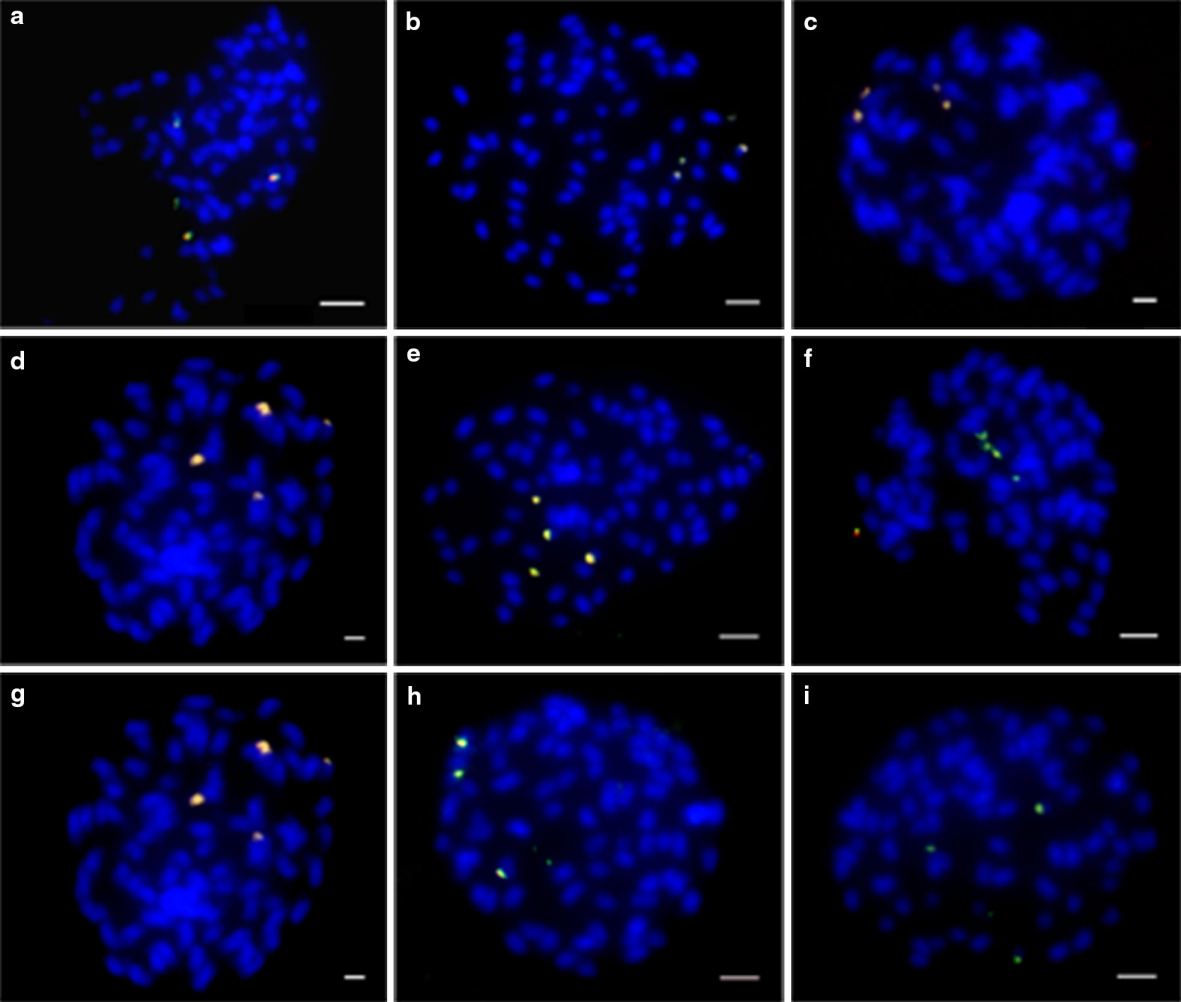
Co-localization of the 3 repeat sequences and 45S rDNA on chromosomes of *Glycine* species

**Fig. 6:**
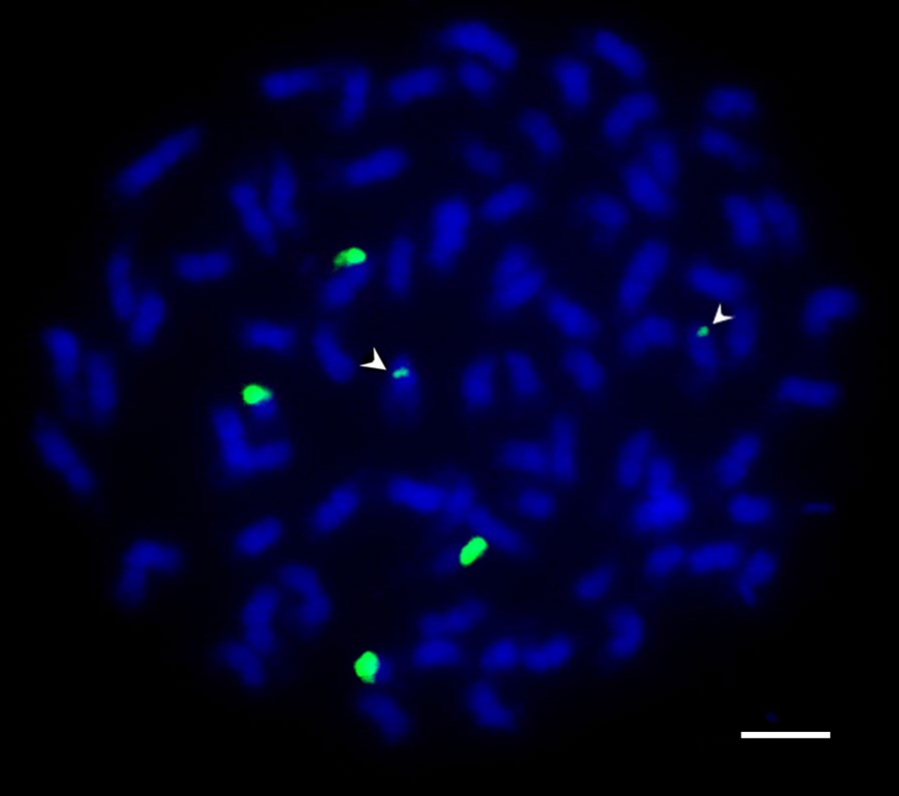
FISH analysis of 45S rDNA on mitotic chromosomes of Tab074

We labeled probes of the three repeat sequences and 45S rDNA in allotetraploid *Glycine* species (i.e., Tom034, Tom039 and Tab 004; Fig. 5), and their diploid relatives (i.e., Tom052, Tom062 and Tom051; Additional file 5: Figure S3; Additional file 6: Figure S4, Additional file 7: Figure S5, Additional file 8: Figure S6). Most of the three tandem repeat sequences signals co-localized with 45S rDNA signals among the tetraploid and diploid relatives tested (Figs. 3, 5; Additional file 5: Figure S3, Additional file 6: Figure S4, Additional file 8: Figure S6). SBRS1 and SBRS3 signals did not co-localize with 45S rDNA signals in the 2 diploid genomes, GG genome (Figs. 2, 4) or DD genome (Additional file 7: Figure S5, Additional file 8: Figure S6). In the genome of Tom062 (DD genome), very weak SBRS1 signals located on many chromosomes (Additional file 7: Figure S5), SBRS2 signals co-localized with 45S rDNA (Additional file 8: Figure S6), and no substantial SBRS3 signal was detected. In addition, all tetraploid accessions showed two 45S rDNA loci, with a minor signal detected in the proximal regions of another pair of chromosomes of Tab 074 (Fig. 6). FISH analysis results are summarized in Table 2.

**Table 2:** Summary of FISH analysis for soybean repeat sequence1 (SBRS1), SBRS2, and SBRS3, 45S rRNA and general ATT repeat

Accessions	Genome	SBRS1	SBRS2	SBRS3	45S	ATT
Soja001	GG	All	2 and 1^a^	3	1	All^b^
Tom052	A_6_A_6_	1^a^	1^a^	1^a^	1	ND
Tom062	DD	Many^c^	Many^a,d^	ND^d^	1	ND
Tom051	D_1_D_1_	1^a^	1^a^	1^a^	1	ND
Tab004	A_6_A_6_B_3_B_3_	2^a^	2^a^	2^a^	2	ND
Tab074	A_6_A_6_B_3_B_3_	2^a^	2^a^	2^a^	3^b^	ND
Tom039	A_6_A_6_DD	2^a^	2^a^	2^a^	2	ND
Tom034	DDD_1_D_1_	2^a^	2^a^	2^a^	2	2–3

One locus has 2 signals on a set of chromosomes, and 2 loci have 4 signals

*ND* No significant signal detected on metaphase chromosome

^a^Signals co-localized with 45S rDNA

^b^2 major and 1 minor signal

^c^Very weak signals

^d^Interphase data were used

### FISH analysis with SSR probe in *Glycine* species

**Fig. 7:**
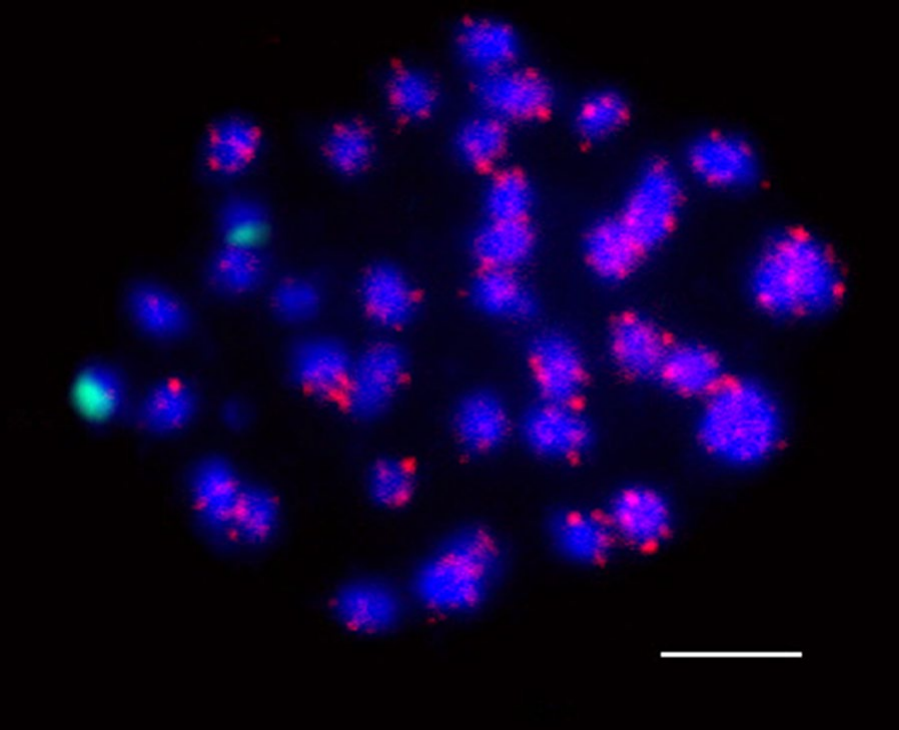
Co-localization of the ATT probe and 45S rDNA on *G. soja* chromosomes

We additionally used 5 types of SSR probes (i.e., ATT, AT, CAA, CT and CTT) in FISH analysis. However, only one SSR probe, with 33 copies of the repeat ATT, on *G. soja*, gave interesting results, and, similar to SBRS1, ATT signals were detected as dispersed signals on every chromosomes of the GG genome (Fig. 7, red). There was no ATT signal on the mitotic chromosomes of most other *Glycine* species except Tom034. Few very weak ATT probe signals were detected on this accession, but the signals were unstable. In addition, the signal distribution for these SSRs varied among chromosomes.

## Discussion

### The predicted repeat sequences were confirmed by cytological study

The soybean WGS sequence project has set significantly new milestones for understanding the soybean genome and its evolutionary history (Jackson et al. 2006; Schmutz et al. 2010). In the current study, we identified three new repeat sequences from soybean WGS data. Using bioinformatics, we propose their characterization in the soybean genome. We used WGS as a resource in chromosome marker design for the cultivated soybean and other *Glycine* species, and found that use of the WGS data was powerful for searching for candidates of repeat sequences and designing probes for cytological research.

For the GG genome, the WGS data could be used to predict general copy numbers of the repeat sequences in each cluster and the location of repeat-sequence condensed regions. FISH analyses confirmed these bioinformatics findings (Figs. 2, 3, 4; Table 2). WGS data may be also used in cytological research for the wild related species. Combining bioinformatics and cytological strategies may help bridge the gap between cultivated species and related species. In the current study, we analysis the three new repeat sequences on many *Glycine* species by FISH. Similar studies had shown that FISH analysis would reveal heterochromatin organization using the centromeric or satellite repeats (Ananiev et al. 1998 for maize; Badaeva et al. 1996 for *Aegilops* species; Kulikova et al. 2004 for *Medicago* species). Because *Glycine* species consist of high chromosome numbers (38, 40, 78, or 80; Hymowitz et al. 1998), as well, these chromosomes are compact and small, the utilization of repetitive sequences as probe for FISH analysis may help on the cytogenesis and breeding analysis in the post-genomics era.

### Possible evolutionary process for the three new repeat sequences among *Glycine* species

Differences in the distribution of repetitive DNA sequences were shown between *Glycine* species in the current study (summarized in Table 2). The chromosomal localization of these DNA probes was different. The possible association of the these repeat sequences and rDNA loci may be due to (1) the three repeat sequences having existed in the rDNA of the genome of their common earlier ancestor, (2) all having expanded and dispersed in GG genome, and finally (3) SBRS1 and SBRS3 being reduced or disappeared in 45S rDNA loci during the GG (Soja001) and DD (Tom062) genome evolution and SBRS2 still existing in the 45S rDNA of all tested *Glycine* species (Table 2). Dolye and his colleague used global, gene-specific, and relaxed clock methods to date the polyploid history of soybean and suggested the date for the *G. max/G. tomentella* split averaged between 3.8 and 6.86 Mya across methods (Egan and Doyle 2010). It coincides well with the fact that the shared repeat sequences, but with different amount of such fragments, among the *Glycine* species.

Many satellite sequences are homologs to the IGS subrepeats of rDNA from other plants genome, such as legumes (Falquet et al. 1997), potato (Stupar et al. 2002), tobacco (Lim et al. 2004; Volkov et al. 1999) and tomato (Jo et al. 2009). In tomato study, the insertion of a retrotransposon into the rDNA gene enhanced the amplification of the nearby IGS subrepeats. Those amplified subrepeats then dispersed into other loci and became repeat sequences (Jo et al. 2009). Other studies also proposed that some transposon elements, such as *En*/*Spm*-like transposons, could be involved in rDNA movement and as one of the key steps when the rDNA subrepeat turned into repeat sequences (Raskina et al. 2004a, 2008; Schubert and Wobus 1985).

### Diversity of the genome in *Glycine* species

We analyzed the DNA content of the nine *Glycine* accessions, five of them are diploid and another four are allotetraploid, by flow cytometry. The two *G. pescadrensis* accessions Tab 004 and Tab 074 collected from Penghu and Kingmen, differed in DNA content (Table 1). In addition, the DNA content of Soja001 and the cultivated soybean (GG genome) differs up to 22 % (Table 1). The DNA content of *G. dolichocarpa* (Tom039) and *G. tomentella* (Tom034) are more than the sum of their two parental diploid species (Table 1).

Research using *Helianthus* (Ungerer et al. 2006) suggested that retrotransposons may play an important role in the genome expansion process of allotetraploid evolution. The dynamics of transposon element driven genome evolution have been reported for several plant genomes (El Baidouri and Panaud 2013). In fact, retrotransposons occupy the largest part of the cultivated soybean genome (Schmutz et al. 2010) and may play a role in *Glycine* genome size variations.

The number of 45S rDNA loci among wild soybeans is dynamic. In previous studies, the number of 45S rDNA loci among the tetraploid *Glycine* species was suggested to be the same (Krishnan et al. 2001; Singh et al. 2001). However, two major and one minor loci of 45S rDNA signals were detected in Tab 074 (*G. pescadrensis*, A_6_A_6_B_3_B_3_ genome). The minor signals are found on the middle area of a pair of the chromosomes. Only two major loci of 45S rDNA were found for the other tetraploids, including Tab 004. These two *G. pescadrensis* accessions were collected from Penghu and Kingmen, and the distance between these two islands is about 140 km, the geographical isolation may explain the diversity of 45S rDNA loci. Thus, the evolution of the 45S rDNA in the *Glycine* genome is more complex than the model of “diploid-doubled to tetraploid”.

Variations in the 45S rDNA loci among natural hybrids and related species were reported for *Lycoris* (Chang et al. 2009), *Oryza* (Chung et al. 2008), *Triticeae* (Kim et al. 1993), and *Arabidopsis* (Maluszynska and Heslopharrison 1993). As well, the ability to alter the number and location of rDNA loci among closely related species or even accessions was noted (Chung et al. 2008; Raskina et al. 2004b). In our study, the variation in number of rDNA loci in *Glycine* was more dramatic than in previous studies (Krishnan et al. 2001; Singh et al. 2001). Such differences in number or location of 45S rDNA may be used as chromosome markers.

### Genome and chromosome markers of *Glycine* species

Specific repeat sequences, such as SBRS1-3, and nonspecific repeat sequences, such as SSR and rDNA, may be potential genome or chromosome markers. The nonspecific repeat sequences can also be used as specific markers, if the distributions of repeats are different enough among genomes. For instance, the SSRs, which are widely identified among species (Varshney et al. 2005), have been used to label the chromosomes of different genomes in hexaploidy *Triticum aestivum* (Cuadrado et al. 2000, 2008). We use nonspecific repeat sequences as probes for labeling chromosomes of several *Glycine* species. Signals of ATT were exclusively detected on the GG genome, with no difference between *G. soja* and *G. max* (data not shown). Although 2–3, weak ATT signals could be detected on the chromosome of Tom034 (data not shown), they were easy to be separated from the strong ones of GG genome.

The study of the *Glycine* genome is important for soybean improvement; combing bioinformatics and cytology techniques can advance investigations. Our study is just a beginning to show that the soybean WSG data and cytological methods can be used to find new repeat sequences, reveal the genome evolution process, and provide addition information in investigation of the phylogeny in the *Glycine* species.

## Additional files

**Additional file 1. Table S1.** Sequence of the primers used in the study.

**Additional file 2. Table S2.** Sequence of repeat sequences and probes used in the study.

**Additional file 3. Figure S1.** Southern blot analysis of SBRS2 on *Glycine* species.

**Additional file 4. Figure S2.** Southern blot analysis of SBRS3 on *Glycine* species.

**Additional file 5. Figure S3.** FISH analysis of SBRS and 45S rDNA on Tom051 chromosomes.

**Additional file 6. Figure S4.** FISH analysis of SBRS and 45S rDNA by FISH on Tom052 chromosomes.

**Additional file 7. Figure S5.** FISH analysis of SBRS1 and 45S rDNA on Tom062 chromosomes.

**Additional file 8. Figure S6.** FISH analysis of SBRS2 and 45S rDNA on Tom062 chromosomes.
