# Supplementary material for: Distribution of new satellites and simple sequence repeats in annual and perennial Glycine species
Source: Bot Stud. 2015 Sep 16;56:22. doi: 10.1186/s40529-015-0103-9 (PMC5430363; doi:10.1186/s40529-015-0103-9)
Supplement: Supplementary file 1 — Additional file 1. Table S1. Sequence of the primers used in the study. [file 40529_2015_103_MOESM1_ESM.doc]

SUPPLEMENTARY TABLE

| Supplementary Table 1. Sequence of the primers used in the study. | | |
| --- | --- | --- |
|  | Forward | Reverse |
| SBRS1 | AGGACGTGTAGTCCTCTGAAGG | ATCAGAGAGGGCTGCACGCCCTCA |
| SBRS2 | GTTGACGCGCGGAGACTA | AAAGGTGCGGGTAACCATAAG |
| SBRS3 | CTCTGCGTGCTATCAGGCTTT | CGTTAACACGCGGAGATTTAC |
| SB92 | GGTCATAACTTTTCACTCGGAG | GAATTTCTCGAGAGCTTCCGTT |
| Satt100 (ATT)33 | ACCTCATTTTGGCATAAA | TTGGAAAACAAGTAATAATAACA |
| Sat_391 (AT) 37 | GCGTAGGCATCGGTCAATATTTT | GCGTTAGCGAGTGGATCAAGATCA |
| BE801128 (CAA)13 | GCGACAGTTCTCCACTCTTC | GCGCCCCTTATAGATTTGTAAC |
| Sct_094 (CT) 20 | GGGTGAAGTGAGAGTAACA | CCCGGATCTCTTCATT |
| Sctt012 (CTT)11 | CCATTATCGCACATCATT | GCTGCATTTTCCCTCTA |

Supplementary Table 2. Sequence of repeat sequences and probes used in the study.

| **SBRS1 monomer** |
| --- |
| TCTCGAGAAATTCAAATGGTCATAACTTTTCACACGGATGTCCGATTCAGGCGCATAATATATCGAGACGCTCGAAATTGAACAACGGAAGC  **SBRS1probe** |
| AGGACGTGTAGTCCTCTGAAGGTGAGGGCGTGCAGCCCTCTGATGGTGAGGACGCGTAGTCCTCTCAAGGCGAGGACGTGTAGTCCTCTGAAGGTGAGGGTAACTAGTACCCAAGGCGAGGGCGGGTAGCCCTCTCAAGGCGAGGACAGGTAGTCCTCTGGAGGTGAGGGCGTGCAGCCCTCTGATAATCAC  **SBRS2 monomer** |
| GTCAACCAGGGGCAAACGAGCCCGTTGACGCGCGGAGACTAACATCGTCTTCTGCACCTTTTGTCGTCCTGACCCGTGAAGTCAGGTGACATGCGGGGTTACCTTATGGTTACCCGCACCTTTC  **SBRS2 probe** |
| AAAGGTGCGGGTAACCATAAGGTAACCCCGTATGTCACCTGACTTCACGGGTCAGGACGAAAAAAGGTGCAGAAGACGATGTTTGTCTCTGCGCGTCAATGGGCTCGTTTGCCCCTGGTTGACGAAAGGTGTGGGTAACCTAAAGGTAACCCTGCATGTCACCTGACTTCACGGGTCAGGACGACAGAAGGTGCAGGAGACGATGTTAGTCTCCGCGCGTCAACAATCAC  **SBRS3 monomer** |
| AGAAGACGACGTTAGTCTCTGCGTGCTATCAGGCTTTTCGTCTTACAGACAGCAAAAAGTTTATACGGATAACCACTCGGGTATTTCCGCCCGTCAGCGTGACTCAAAAGTCAGTATGACAGATCTTGTGAGCGCGGAAGATGACGTAAATCTCCGCGTGTTAACGGGCTTGTCGGCCGCGATTGACGAAGGGCGC  **SBRS3 probe** |
| AAGCCTGATAGCACGCAGAGACTAACGTCGTCTTCTGCGCCCTTCGTCAATCGCGGCCGACATGCCCATTGACACATGGAGATTTACGTTATCTTCCGCGCTCACAAGATCTGTCATACTGACTTTTGAGTCACGCTGACGGGCGGAAATACCCGAGTGGTTATCCGTATAAACATTATTTTTTGCTGTCTGTAAGACGAAAAGCCTGATAGCACGCAGAGACTAACGTCGTCTTCTGCGCCCTTCGTCAATCGCGGCCGACAAGCCCGCTGACACGCGGAGATTTACGTCATCTTCCGCGCTCACAAGATCTGTCACACTGACATTTGAGTCACGCTGACGGGCGGAAATACCCCGAGTGGTTATCCGAATCAC  **ATT probe** |
| GCGTAATTAACACCAATATATGACATG (ATT)_33_ ACATTTTGAATTTTTAACCCCGC  **AT probe** |
| GCGTAGGCATCGGTCAATATTTT (AT)_37_ TGATCTTGATCCACTCGCTAACGC  **CAA probe** |
| GCGACAGTTCTCCACTCTTC (CAA)_13_ GTTACAAAVCVATAAGGGGCGC  **CT probe** |
| GGGTGAAGTGAGAGTAACA (CT)_20_ AATGAAGAGATCCGGG  **CTT probe** |
| CCATTATCGCACATCATT (CTT)_11_ TAGAGGGAAAATGCAGC |
