# Supplementary material for: Distribution of new satellites and simple sequence repeats in annual and perennial Glycine species
Source: Bot Stud. 2015 Sep 16;56:22. doi: 10.1186/s40529-015-0103-9 (PMC5430363; doi:10.1186/s40529-015-0103-9)
Supplement: Supplementary file 2 — Additional file 2. Table S2. Sequence of repeat sequences and probes used in the study. [file 40529_2015_103_MOESM2_ESM.pdf]

Supplementary Table 2. Sequence of repeat sequences and probes used in the study.

SBRS1 monomer

TCTCGAGAAATTCAAATGGTCATAACTTTTCACACGGATGTCCGATTCAGGCGCATAATATATCGAGACGCTCGAAA
TTGAACAACGGAAGC

SBRS1probe

AGGACGTGTAGTCCTCTGAAGGTGAGGGCGTGCAGCCCTCTGATGGTGAGGACGCGTAGTCCTCTCAAGGCGAGG
ACGTGTAGTCCTCTGAAGGTGAGGGTAACTAGTACCCAAGGCGAGGGCGGGTAGCCCTCTCAAGGCGAGGACAGG
TAGTCCTCTGGAGGTGAGGGCGTGCAGCCCTCTGATAATCAC

SBRS2 monomer

GTCAACCAGGGGCAAACGAGCCCGTTGACGCGCGGAGACTAACATCGTCTTCTGCACCTTTTGTCGTCCTGACCCG
TGAAGTCAGGTGACATGCGGGGTTACCTTATGGTTACCCGCACCTTTC

SBRS2 probe

AAAGGTGCGGGTAACCATAAGGTAACCCCGTATGTCACCTGACTTCACGGGTCAGGACGAAAAAAGGTGCAGAAG
ACGATGTTTGTCTCTGCGCGTCAATGGGCTCGTTTGCCCCTGGTTGACGAAAGGTGTGGGTAACCTAAAGGTAACC
CTGCATGTCACCTGACTTCACGGGTCAGGACGACAGAAGGTGCAGGAGACGATGTTAGTCTCCGCGCGTCAACAA
TCAC

SBRS3 monomer

AGAAGACGACGTTAGTCTCTGCGTGCTATCAGGCTTTTCGTCTTACAGACAGCAAAAAGTTTATACGGATAACCACT
CGGGTATTTCCGCCCGTCAGCGTGACTCAAAAGTCAGTATGACAGATCTTGTGAGCGCGGAAGATGACGTAAATCT
CCGCGTGTTAACGGGCTTGTCGGCCGCGATTGACGAAGGGCGC

SBRS3 probe

AAGCCTGATAGCACGCAGAGACTAACGTCGTCTTCTGCGCCCTTCGTCAATCGCGGCCGACATGCCCATTGACACA
TGGAGATTTACGTTATCTTCCGCGCTCACAAGATCTGTCATACTGACTTTTGAGTCACGCTGACGGGCGGAAATACC
CGAGTGGTTATCCGTATAAACATTATTTTTTGCTGTCTGTAAGACGAAAAGCCTGATAGCACGCAGAGACTAACGTC
GTCTTCTGCGCCCTTCGTCAATCGCGGCCGACAAGCCCGCTGACACGCGGAGATTTACGTCATCTTCCGCGCTCAC
AAGATCTGTCACACTGACATTTGAGTCACGCTGACGGGCGGAAATACCCCGAGTGGTTATCCGAATCAC

ATT probe

GCGTAATTAACACCAATATATGACATG (ATT)$_{33}$ ACATTTTGAATTTTTAACCCCGC

AT probe

GCGTAGGCATCGGTCAATATTTT (AT)$_{37}$ TGATCTTGATCCACTCGCTAACGC

CAA probe

GCGACAGTTCTCCACTCTTC (CAA)$_{13}$ GTTACAAAVCVATAAGGGGCGC

CT probe

GGGTGAAGTGAGAGTAACA (CT)$_{20}$ AATGAAGAGATCCGGG

CTT probe

CCATTATCGCACATCATT (CTT)$_{11}$ TAGAGGGAAAATGCAGC
