# Supplementary figures and images for: Distribution of new satellites and simple sequence repeats in annual and perennial Glycine species
Source: Bot Stud. 2015 Sep 16;56:22. doi: 10.1186/s40529-015-0103-9 (PMC5430363; doi:10.1186/s40529-015-0103-9)

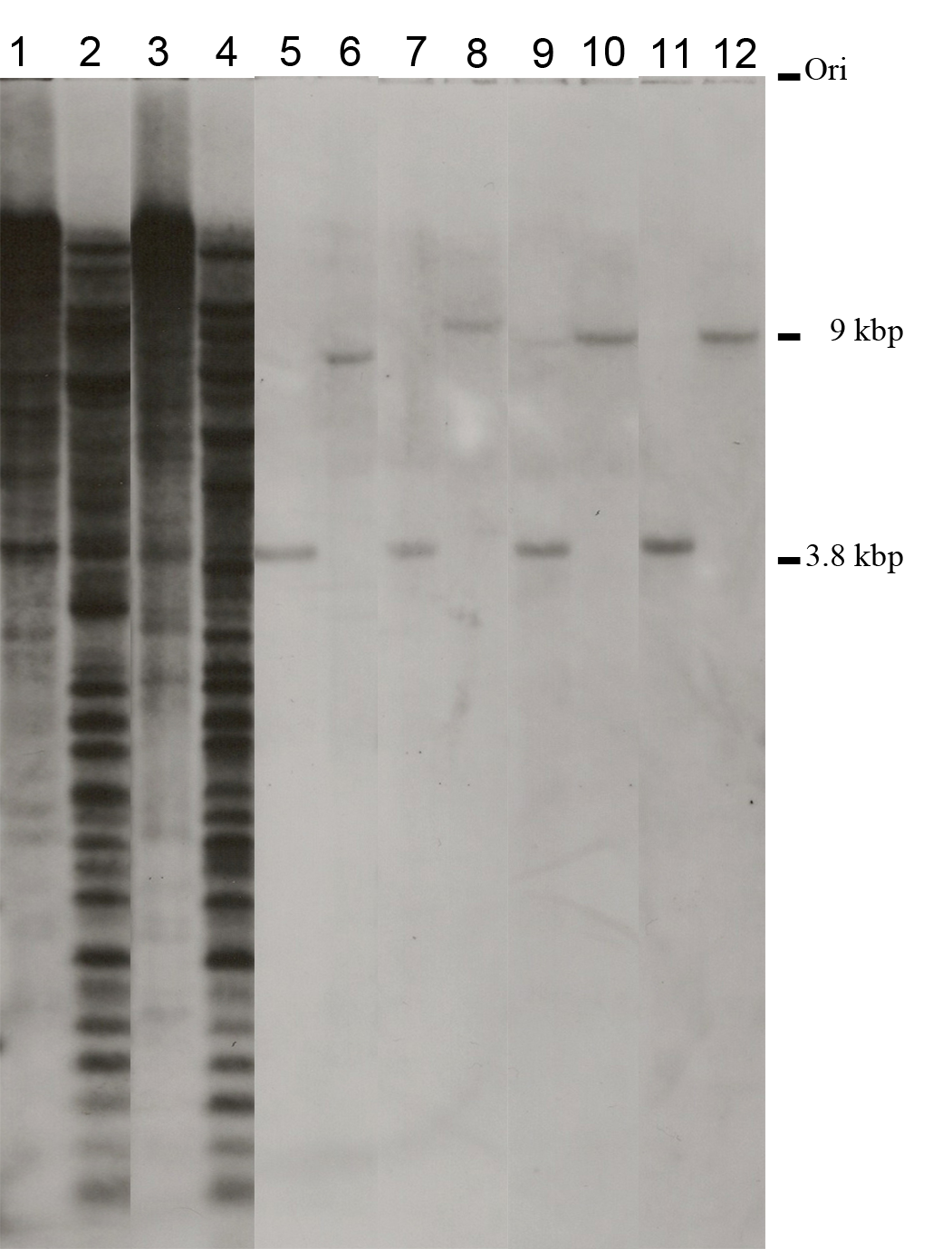

Supplement: Supplementary file 3 — Additional file 3. Figure S1. Southern blot analysis of SBRS2 on Glycine species. [file 40529_2015_103_MOESM3_ESM.tiff]

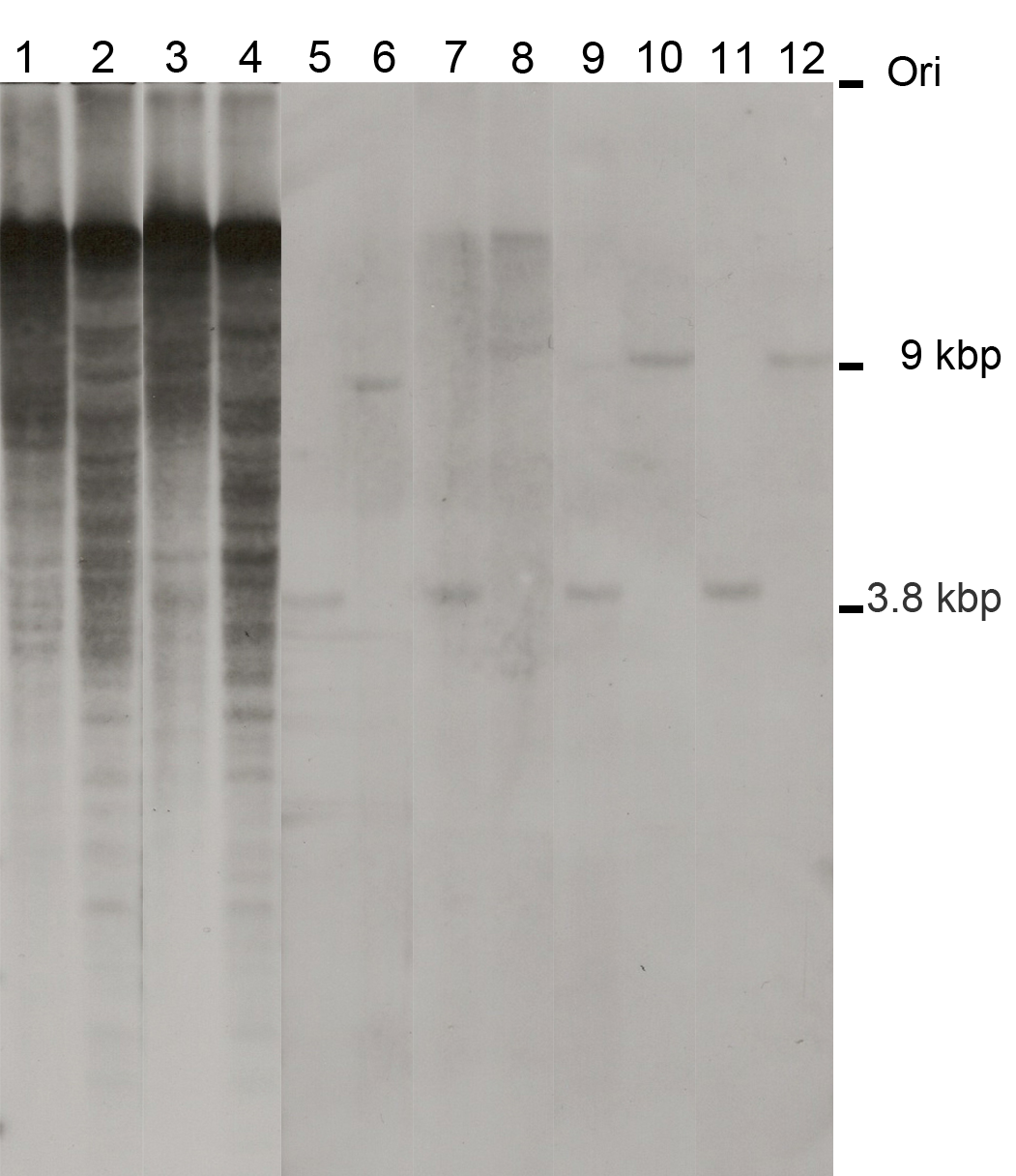

Supplement: Supplementary file 4 — Additional file 4. Figure S2. Southern blot analysis of SBRS3 on Glycine species. [file 40529_2015_103_MOESM4_ESM.tiff]

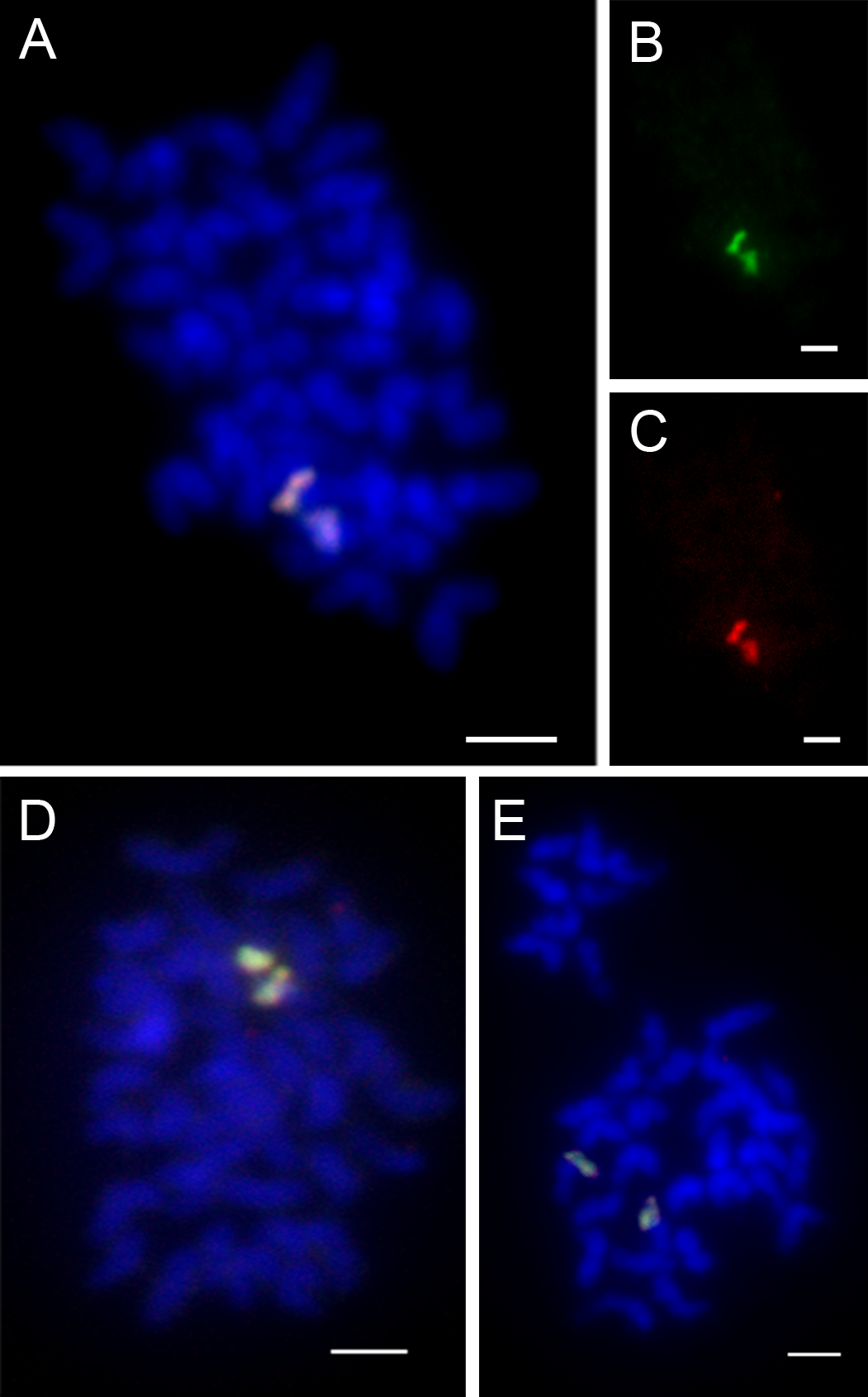

Supplement: Supplementary file 5 — Additional file 5. Figure S3. FISH analysis of SBRS and 45S rDNA on Tom051 chromosomes. [file 40529_2015_103_MOESM5_ESM.tiff]

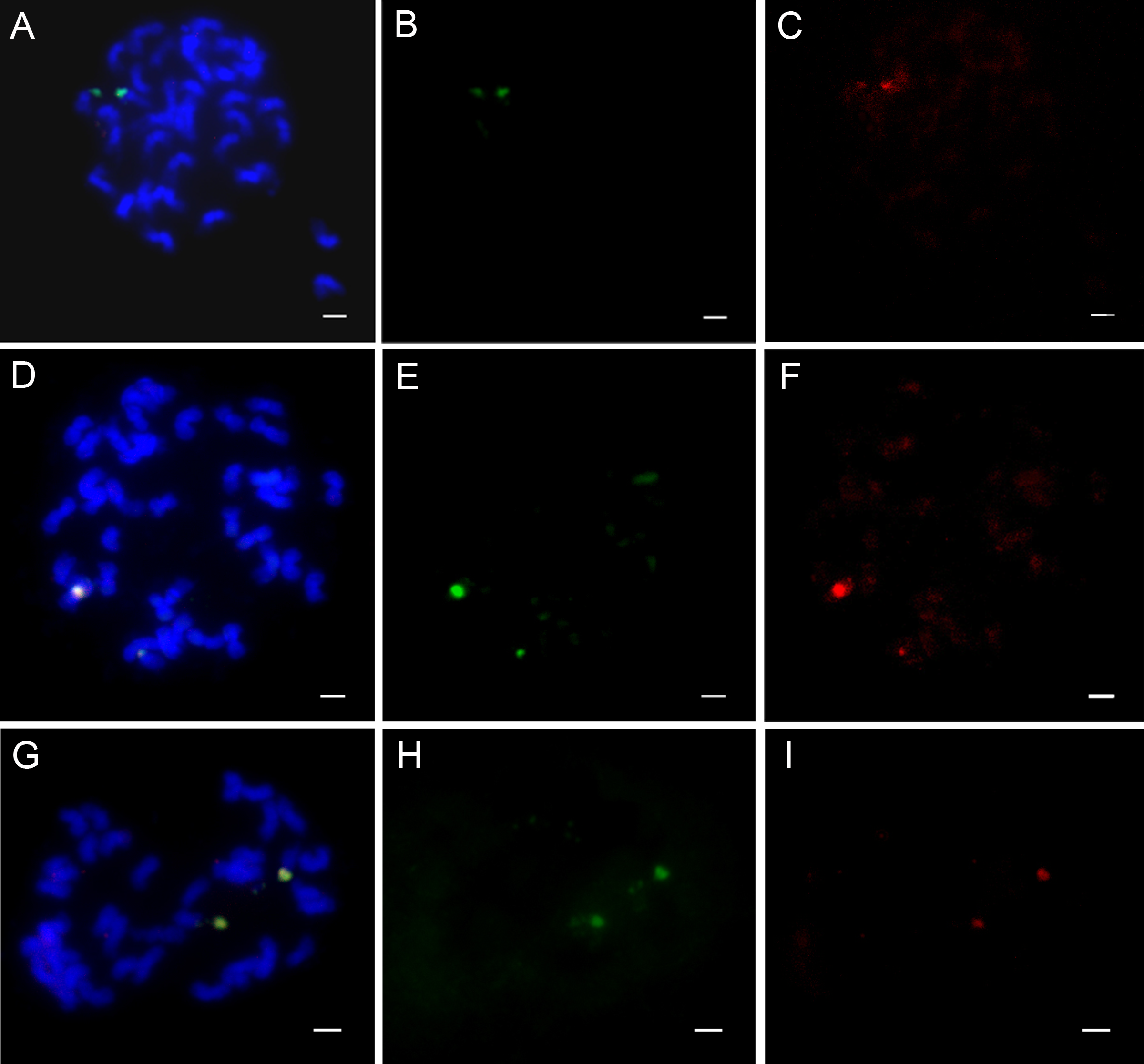

Supplement: Supplementary file 6 — Additional file 6. Figure S4. FISH analysis of SBRS and 45S rDNA by FISH on Tom052 chromosomes. [file 40529_2015_103_MOESM6_ESM.tiff]

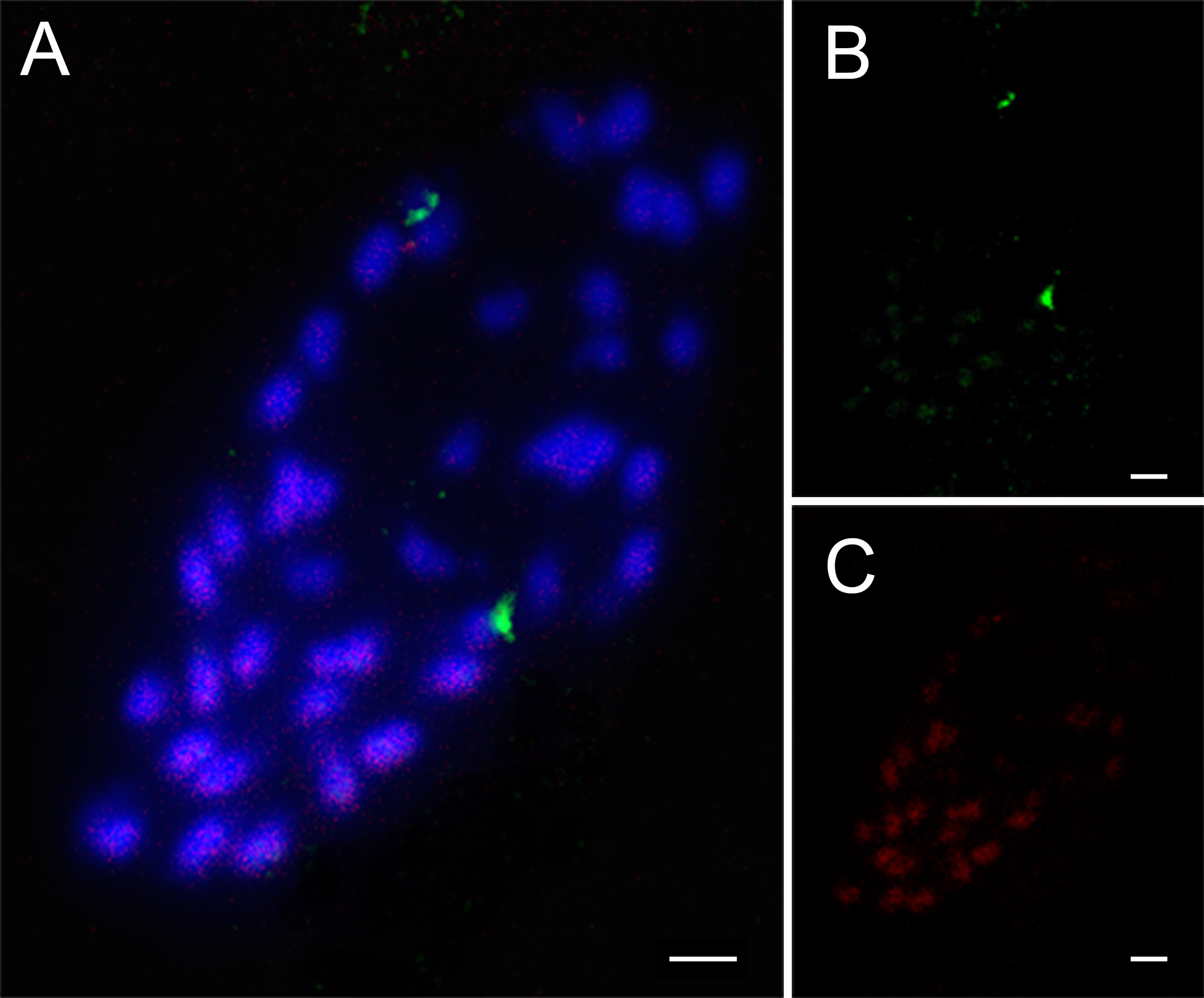

Supplement: Supplementary file 7 — Additional file 7. Figure S5. FISH analysis of SBRS1 and 45S rDNA on Tom062 chromosomes. [file 40529_2015_103_MOESM7_ESM.tiff]

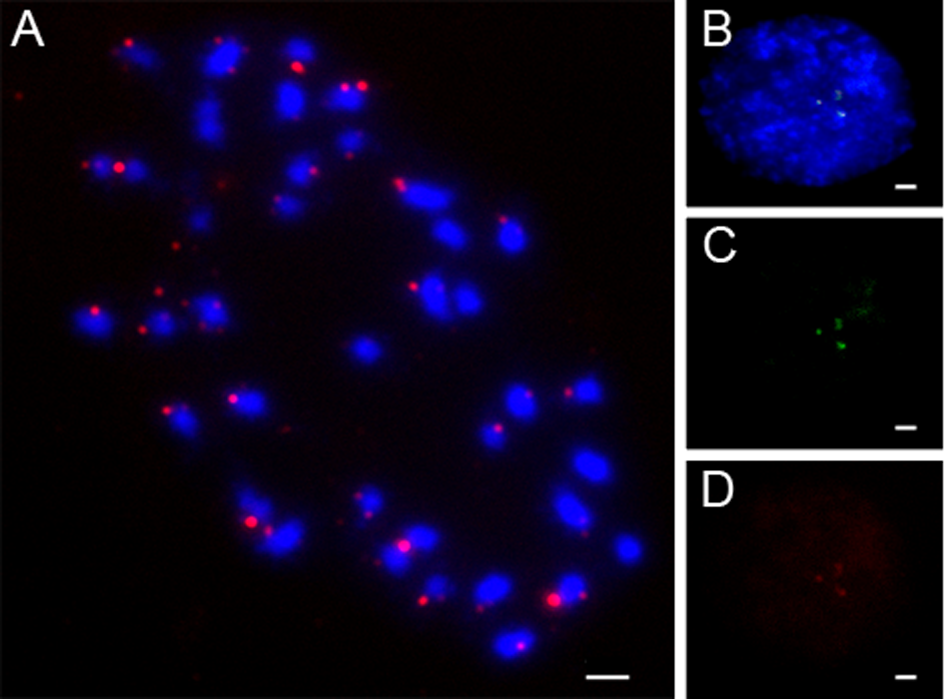

Supplement: Supplementary file 8 — Additional file 8. Figure S6. FISH analysis of SBRS2 and 45S rDNA on Tom062 chromosomes. [file 40529_2015_103_MOESM8_ESM.tiff]
